# Pluripotent Stem Cell-Derived Hepatocytes Phenotypic Screening Reveals Small Molecules Targeting the CDK2/4-C/EBPα/DGAT2 Pathway Preventing ER-Stress Induced Lipid Accumulation

**DOI:** 10.3390/ijms21249557

**Published:** 2020-12-15

**Authors:** Maddalena Parafati, Sang Hyo Bae, R. Jason Kirby, Martina Fitzek, Preeti Iyer, Ola Engkvist, David M. Smith, Siobhan Malany

**Affiliations:** 1 Department of Pharmacodynamics, College of Pharmacy, University of Florida, Gainesville, FL 32610, USA; mparafati@cop.ufl.edu (M.P.); sarahbae97_1@outlook.com (S.H.B.); 2 Conrad Prebys Center for Chemical Genomics, Sanford Burnham Prebys Medical Discovery Institute, La Jolla, CA 92037, USA; jasonkirby62@yahoo.com; 3 Hit Discovery, Discovery Sciences, R&D, AstraZeneca, Alderley Park, Macclesfield SK10 4TG, UK; Martina.Fitzek@astrazeneca.com; 4 Molecular AI, Discovery Sciences, R&D, AstraZeneca, 431 83 Mölndal, Sweden; preeti.iyer1@astrazeneca.com (P.I.); ola.engkvist@astrazeneca.com (O.E.); 5 Emerging Innovations Unit, Discovery Sciences, R&D, AstraZeneca, Cambridge SG8 6HB, UK; dave.smith@astrazeneca.com

**Keywords:** human induced pluripotent stem cell-derived hepatocytes, chemogenomic library screening, quantitative high-content microscopy analysis, lipid droplets, steatosis, NAFLD, drug discovery, open innovation, CDK4-C/EBPα/DGAT2 pathway

## Abstract

Non-alcoholic fatty liver disease (NAFLD) has a large impact on global health. At the onset of disease, NAFLD is characterized by hepatic steatosis defined by the accumulation of triglycerides stored as lipid droplets. Developing therapeutics against NAFLD and progression to non-alcoholic steatohepatitis (NASH) remains a high priority in the medical and scientific community. Drug discovery programs to identify potential therapeutic compounds have supported high throughput/high-content screening of in vitro human-relevant models of NAFLD to accelerate development of efficacious anti-steatotic medicines. Human induced pluripotent stem cell (hiPSC) technology is a powerful platform for disease modeling and therapeutic assessment for cell-based therapy and personalized medicine. In this study, we applied AstraZeneca’s chemogenomic library, hiPSC technology and multiplexed high content screening to identify compounds that significantly reduced intracellular neutral lipid content. Among 13,000 compounds screened, we identified hits that protect against hiPSC-derived hepatic endoplasmic reticulum stress-induced steatosis by a mechanism of action including inhibition of the cyclin D3-cyclin-dependent kinase 2-4 (CDK2-4)/CCAAT-enhancer-binding proteins (C/EBPα)/diacylglycerol acyltransferase 2 (DGAT2) pathway, followed by alteration of the expression of downstream genes related to NAFLD. These findings demonstrate that our phenotypic platform provides a reliable approach in drug discovery, to identify novel drugs for treatment of fatty liver disease as well as to elucidate their underlying mechanisms.

## 1. Introduction

Non-alcoholic fatty liver disease (NAFLD) encompasses a progressive disease spectrum which ranges in severity from excessive accumulation of fat deposition in the liver through infiltration along with liver inflammation (non-alcoholic steatohepatitis, NASH), to fibrosis and cirrhosis. Approximately 25% of patients with cirrhosis will develop hepatocellular carcinoma (HCC) [1,2]. The progression of NAFLD, considered to be among the most common liver diseases world-wide [3,4] with a global prevalence of about 24% [5], is highly variable among individuals. A minority of individuals progress to end-stage liver disease and/or develop HCC [6]. Such inter-individual variability, coupled with a lack of understanding of the underlying mechanisms, has limited the development of effective therapeutic interventions.

Managing fat accumulation in the liver is considered an important way to prevent or treat NAFLD. However, the effective therapeutic options for treating NAFLD are limited [7]. The primary causes of the disease are various and complex and include genetic predisposition, metabolic syndrome, obesity, type II diabetes, insulin resistance, drugs and physical inactivity [8,9,10,11,12]. The pathophysiology of NAFLD involves impaired hepatocyte metabolism, in particular because of excessive fatty acid uptake [13]. Other potential pathogenic mechanisms include decreased fatty acid oxidation, exaggerated de novo lipogenesis or retention of lipids due to reduced very low density lipoprotein synthesis and impaired hepatocyte apolipoprotein secretion by hepatocyte and endoplasmic reticulum stress [13,14,15,16,17]. Thus, human in vitro models of hepatic steatosis with fat accumulation that reflect molecular processes underlying the etiology of NAFLD for the discovery of new therapeutics are needed.

Human hepatocyte-like cells have been proposed as an inexhaustible source of hepatocytes for modeling NAFLD [18,19,20,21,22]. Specifically, human-induced pluripotent stem cell-derived hepatocytes (hiPSC-Hep) have similar biological properties to primary liver cells. However, hiPSC-Hep remain difficult to maintain for extended culture times and do not yet display a fully mature phenotype to model chronic liver disease. Nonetheless, hiPSC-Hep provide a stable, readily available cell source for applications including the investigation of defects in lipid metabolism [23,24], protein accumulation [25], mitochondrial defects [26,27], and toxicity screening [28,29,30] and may represent a physiologically relevant model system for drug screening [31,32,33]. In fact, hiPSC-Hep derived from both healthy donors and patients with liver disease can reproduce a steatosis cell stage resembling NAFLD to investigate the molecular mechanisms underlying liver disease onset and progression. [33,34,35].

Previously, we developed and characterized a phenotypic assay platform using hiPSC-Hep to trigger endoplasmic reticulum (ER) stress-induced steatosis in the cells and synergistically promote triglyceride accumulation and gene dysregulation involved in fatty acid and triglyceride biosynthesis [34]. Transcriptomic analysis revealed upregulation of genes in the ER stress-unfolded protein response (UPR) axis including stanniocalcin-2 (STC2), a pro-survival component, and fibroblast growth factor 21 (FGF21), a marker of hepatic fat, targets that are increased in NASH patients [36]. Reversal of lipid accumulation and gene expression was achieved by treatment of hiPSC-Hep with obeticholic acid (OCA), a farnesoid X receptor (FXR) agonist and clinical candidate for NASH.

Our 384-well assay high content platform is amenable to high throughput screening (HTS) to identify compounds that reduce the steatosis phenotype. hiPSC-Hep technology in combination with HTS has led to the discovery of novel therapeutics for the treatment of congenital liver diseases [23,25,27]. In this manuscript, we highlight the potential of hiPSC-Hep as a means to model human liver steatosis under ER stress, recapitulating in vitro disease-specific phenotype and utilize this approach in combination with multiplexed high content screening for the identification of small molecule inhibitors of cellular lipid deposition. In collaboration with AstraZeneca’s Open Innovation initiative, we screened 13,000 annotated molecules including compounds with high affinity targeting approximately 1763 human proteins.

Our top hits that confirmed dose-dependent inhibition of lipid droplet accumulation included cyclin D3-cyclin-dependent kinase (CDKs) inhibitors. CDK2 and CDK4 are considered potential targets for prevention and treatment of hepatic steatosis [37,38]. Other studies suggested that inhibition of CDK4 plays an important role in hepatic fatty acid metabolism in reducing CCAAT-enhancer-binding proteins (C/EBPα)/ histone acetyltransferase p300 complexes and eliminating hepatic steatosis [39]. Our confirmed hits were further validated by target gene-expression studies to determine what pathways were altered by these molecules. Our results suggest that inhibition of the hiPSC-Hep steatosis phenotype involves the CDK-C/EBPα axis. In conclusion, these CDK inhibitors, identified by a hiPSC-Hep high content screening approach may help elucidate underlying disease mechanisms of NAFLD and may form the basis for developing new therapeutic agents for slowing or preventing NAFLD progression.

## 2. Results

### 2.1. Validation of a Phenotypic High-Content Assay for ER-Stress induced Lipid Accumulation

A monolayer of hiPSC-Hep, with hepatocyte-like morphology, gene expression and function, was used as a cell model for induction of steatosis and high-content analysis to detect neutral lipid droplet accumulation and inhibition. We showed active uptake of free fatty acids from an exogenous source of oleic and palmitic acid and subsequent conversion to triglycerides (TAG) stored as lipid droplets (Supplemental Figure S1A,B). Triacsin C, an inhibitor of the de novo synthesis of triglycerides by blocking long fatty acid acyl-CoA synthesis, fully inhibits oleic acid-induced lipid droplet accumulation predominately mediated via diacylglycerol O-acyltransferase 2 (DGAT2). Triacsin C did not inhibit palmitic acid-induced lipid accumulation suggesting multiple pathways promoting lipid storage are active in our model [35]. Incubation of the cells with a saturated fluorescently labeled fatty acid analog confirmed localization into neutral lipid vacuoles (Supplementary Figure S1C–F).

**Figure 1:**
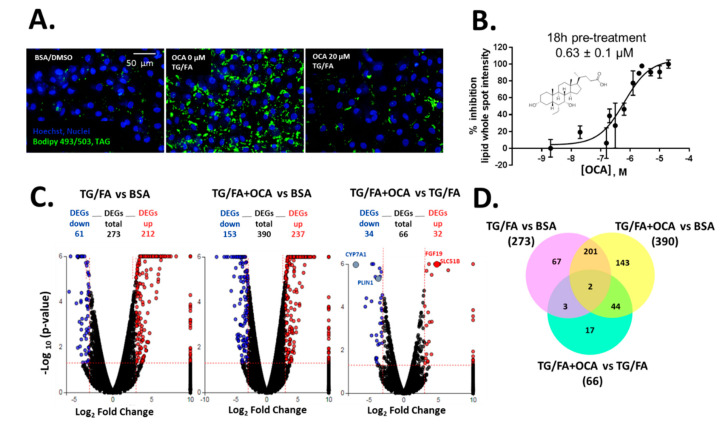
Human-induced pluripotent stem cell-derived hepatocytes (hiPSC-Hep) lipid accumulation assay validation with obeticholic acid (OCA) (**A**) Representative images of lipid droplet-stained cells show exposure to OCA at 20 µM in thapsigargin and free fatty acid mixture (TG/FA)-treated cells reduced lipid content to control levels (BSA-treated cells) compared with TG/FA only treated cells. (**B**) OCA dose-dependently reduced phenotypic readout in A. Overview of the transcriptome data. (**C**) Volcano plots of differentially expressed genes (DEGs), with fold-change over ± 3.0 (blue: down-regulated, and red: up-regulated genes) and *p*-value < 0.05, between TG/FA vs. BSA, TG/FA+OCA vs. BSA and TG/FA+OCA vs TG/FA. The dotted line in red marks the gene expression fold-change (Log_2_ fold change) on the X-axis versus statistical significance (-Log10 (*p*-value)) on the Y-axis. The number of DEGs for each treatment group are indicated. FXR target genes are labeled in the rightmost volcano plot. (**D**) Venn diagram showing the overlap of DEGs between each set of genes across the treatment groups TG/FA vs. BSA, TG/FA+OCA vs. BSA and TG/FA+OCA vs. TG/FA and represents the DEGs that passed the cutoff of a fold-change of at least ±3 based on RNA-seq data.

We previously showed that treatment with thapsigargin in addition to the free fatty acid cocktail induced crosstalk between endoplasmic reticulum (ER)-uncoupled protein response regulatory network and de novo lipogenesis [34]. The phenotypic assay was measured and quantified by BODIPY 493/503-stained neutral lipid droplets multiplexed with Hoechst dye to detect viable cell count, as shown by the process in Supplementary Figure S2. Obeticholic acid (OCA), a clinically advanced therapeutic and farnesoid X receptor (FXR) agonist evaluated in phase III trials for the treatment of NASH [40], was used as an inhibitor control of lipid accumulation (Figure 1A). We found that OCA reduced lipid accumulation dose-dependently with a half maximal inhibitory concentration (IC_50_) of 0.63 µM (pIC_50_ = 6.2 ± 0.3) when cells were pre-treated for 24 h and co-treated for an additional 18 h in the presence of thapsigargin and free fatty acid mixture (TG/FA) (Figure 1B). Our results are in accordance with therapeutic plasma concentrations of OCA (0.290 µM) [41] and with studies performed in a human liver in vitro system (0.5 µM) [42]. Transcriptomic analysis showed 273 and 390 differentially expressed genes (DEGs) arising from exposure of treated hiPSC-Hep to TG/FA and TG/FA plus OCA compared to control levels, respectively, and only 66 DEGs corresponding to the reversal in the phenotype due to OCA pre-incubation of TG/FA-treated cells as determined by iPathwayGuide functional annotation analysis (Figure 1C). The minimum number of DEGs was observed with TG/FA+OCA vs. TG/FA treatment groups (Figure 1C, rightmost volcano plot). In contrast, the maximum number of DEGs was observed with TG/FA+OCA vs. BSA treatment groups (Figure 1C, middle volcano plot). These results indicate a rapid organization of the transcriptome in response to OCA challenge when compared to disease model (TG/FA) or control (BSA), respectively. FXR target genes, including fibroblast growth factor 19 (FGF19), solute carrier family 51 subunit Beta (SLC51B), cytochrome P450 7A1 (CYP7A1) and perilipin 1 (PLIN1) are regulated by OCA under our treatment conditions. We previously showed the top Kyoto Encyclopedia of Genes and Genomes (KEGG) pathways linked to genes implicated in bile acid secretion and disposal after OCA-TG/FA treatment [34].

DEGs were also compared using a Venn diagram. There were 67 out of 273 DEGs in TG/FA vs. BSA; 143 out of 190 DEGs TG/FA+OCA vs. BSA; 17 out of 66 DEGs in TG/FA+OCA vs. TG/FA. These results indicated that after selective activation by OCA, FXR may activate the gene expression of 17 key genes in hiPSC-Hep under ER stress (Figure 1D and Supplemetary Excel spreadsheets). Based on our validation studies with OCA, we proceeded to treat cells with OCA or the test compound prior to treatment with TG/FA, as opposed to co- or post-treatment as a standard primary approach for drug screening to discover new potential inhibitors of ER-stress induced lipid accumulation.

### 2.2. Screening of an Annotated Chemical Library Confirmed Kinase Hits as Lipid Droplet Inhibitors

**Figure 2:**
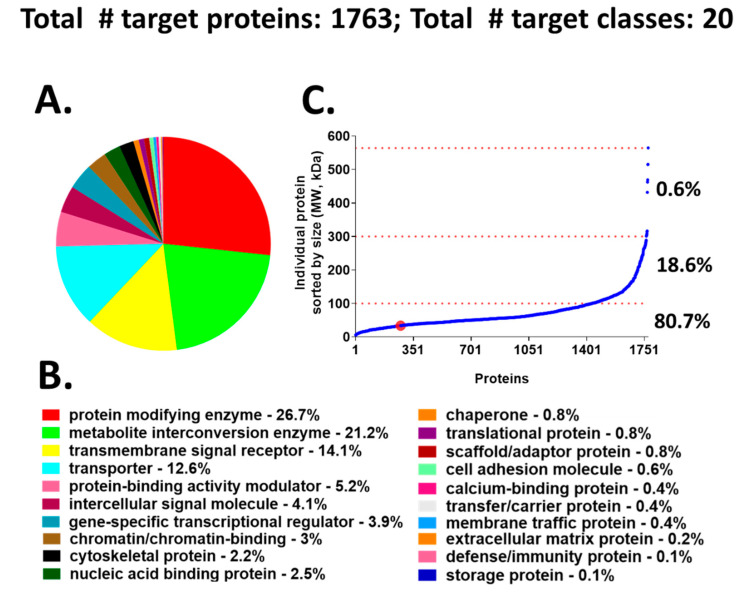
Compound library target classes. (**A**) A graphical overview of 1763 compound–target protein coverage converted into functional classification by the Panther classification system. (**B**) The 20 target classes and relative percentage of each functional class shown in A. (**C**) Molecular size-distribution of individual target proteins fall into three groups with relative percentage reported on the right. The red dot identifies the cyclin dependent kinase hits from the screen; these hits are incorporated in the protein modifying enzyme class (red).

We chose to use a target-annotated compound library, supplied through a collaboration with AstraZeneca’s Open Innovation Group (https://openinnovation.astrazeneca.com), to screen in our validated hiPSC-Hep phenotypic assay. This approach provided a target reference for classifying the hits we identified by HTS. The library consists of bio-annotated, mainly chemical leads in the public domain that impact several signaling pathways and covers 1763 known molecular targets classified into 20 functional protein classes (Figure 2A,B). The target protein classes organized by protein size, align into the following three groups: 6 to 100 kDa (~80.7%), 101 to 300 kDa (~18.6%), and >300 kDa (0.6%) (Figure 2C). The advantage of screening an annotated library in our phenotypic cellular assay is that compound targets can be identified quickly after hit confirmation and may reveal novel biological mechanisms for compounds whose annotation is driving the phenotypic effect.

**Figure 3:**
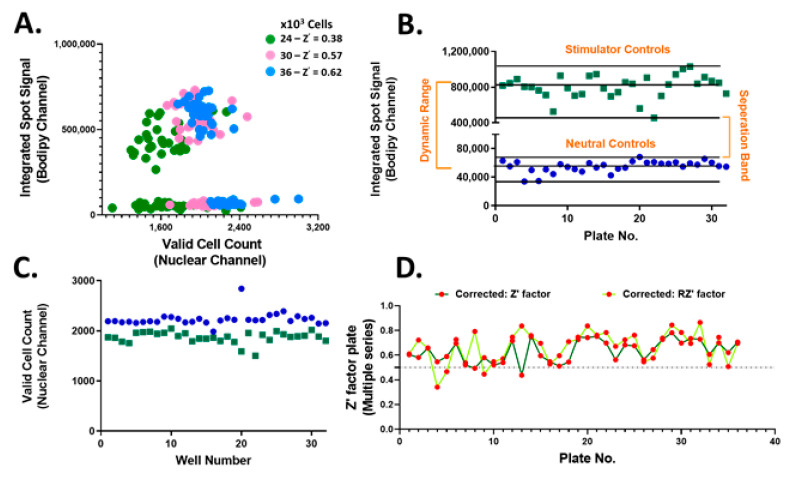
High-throughput screening performance of bioactive compounds in hiPSC-Hep. (**A**) Integrated spot signal (ISS) vs. valid cell count in 2D scatter plot for hiPSC-Hep seeded at 24K (green, Z’ = 0.38), 30K (pink, Z’ = 0.57) and 36K (blue, Z’ = 0.62) per well cell densities. Average of TG/FA treated wells (green squares) and average of BSA-treated cells (blue dots) per screening plate for (**B**) ISS relative values of BODIPY 493/503 fluorescence imaged in the green channel and (**C**) valid cell count relative to values of Hoechst 33342 fluorescence detected in the blue channel. Each data point represents the mean relative ISS from six fields per well and the average of 16 wells per plate. (**D**) Determined Z’ scores (dark green line) and robust Z’ scores (RZ’ light green line) calculation for ISS values in TG/FA vs. BSA treated controls per assay plate.

The accumulation of neutral lipids is an important parameter for lipid homeostasis. Thus, the neutral lipid whole cell staining intensity which is directly proportional to the per cell total neutral lipid content, referred to as the integrated spot signal, was measured and applied as a screening criterion. In addition, various parameters to the Hoechst nuclear staining including size, shape and brightness were applied to identify valid nuclei. Optimal hiPSC-Hep seeding density was determined to be 36,000 cells/well chosen based on the resulting Z’ (Figure 3A). These results confirmed the suitability of implementing treated hiPSC-Hep cells for HTS and pre-treatment with test compounds. Using this platform, we screened 13,000 bioactive compounds covering the target classes shown in Figure 2 in single point at 10 μM concentration in 384-well plates. We monitored inhibition of lipid droplet accumulation by using TG/FA-treated hiPSC-Hep as the stimulator control, column 11 on each screening plate for maximal lipid accumulation and BSA-treated hiPSC-Hep as the neutral control, column 12 on each screening plate, in which no lipid droplet accumulation occurred. We developed an automated high content image analysis algorithm to each screening plate to detect an integrated spot signal imaged in the green channel which depicts BODIPY 493/503 stained lipid droplets multiplexed with valid cell count in the nuclei channel, as described in Materials and Methods. The raw screening data visualized using GeneData software is shown in Supplemental Figure S3. The distribution of the lipid staining in hiPSC-Hep was consistent across 36 × 384-well plates with a large dynamic range and no significant effects on valid cell count (Figure 3B,C and Figure S4). The assay exhibited excellent robustness with Z’ values between 0.43 and 0.78 for negative and positive controls, respectively (Figure 3D).

**Figure 4:**
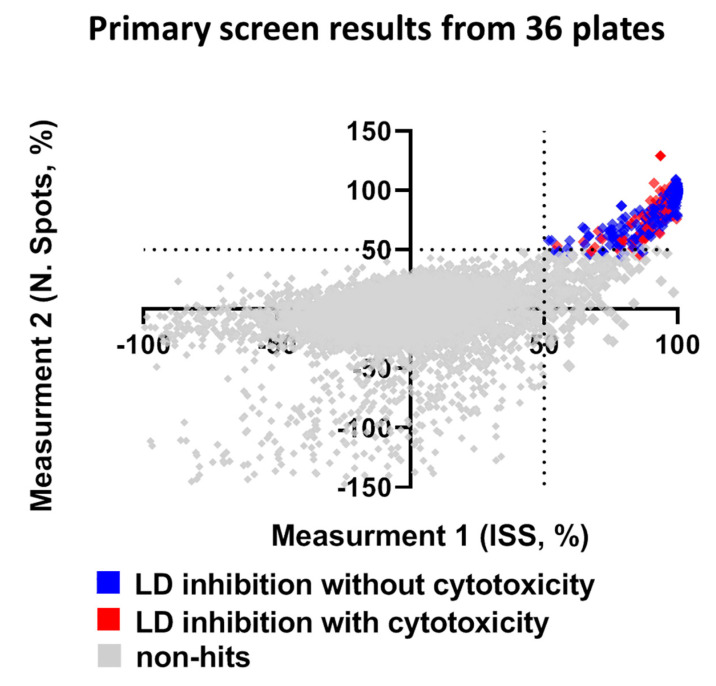
Summary of library screen and hit confirmation. The scatter plot for ISS versus number of spots after normalization in non-treated hiPSC-Hep indicates wells with no effect (grey squares), hits as ≥ 50% ISS and N. Spots and >60% valid cell count (blue squares) and cytotoxic as ≤40% valid cell count (red squares). Each dot represents the mean of six fields from one well treated with one compound from the library.

Compounds that reduced the integrated spot signal ≥ 50% without severe cytotoxicity (valid cell count >60% of the average cell count of control wells) were marked as hits in the primary screen. Application of this criteria to HTS resulted in a total of 158 compounds that caused significant lipid droplet reduction after filtering out compounds that were cytotoxic to hiPSC-Hep (Figure 4 and Supplementary Figure S4). Hit compounds were replenished as fresh stocks and evaluated in the same assay platform in triplicate across three different plates at both 10 μM and 1 μM to confirm primary activity and evaluate for dose-dependent response. Of the 158 compounds retested, 20% were confirmed hits that significantly reduced TAG dose-dependently at the two concentrations and less than 2% were cytotoxic. While our reconfirmation rate was low, 29 compounds, readily available from AstraZeneca’s compound stocks were confirmed as hits in the primary screen at 10 μM, did not show significant effect on valid cell count and showed activity at 1 μM.

### 2.3. Cyclin-Dependent Kinase Inhibitors as Modulators of Hepatic Fat

Confirmed hits were resupplied as DMSO stocks and tested in a 10-point dose-response using the same protocol for the multiplexed phenotypic HTS platform. Of the 29 compounds, 21 showed a dose-dependent inhibition of lipid accumulation with IC_50_ ≤ 10 μM. Nonlinear curve fit analyses of these compounds are shown in the Supplementary Material (Figure S5). From the library database, we extracted activity annotations (pXC_50_) to obtain the compound hit target profiles. Since the library consists of drug-like compounds with target activities in the range of 7.0–10.0, an activity cut-off of pXC_50_ = 7.0 was used to highlight the key targets for the prioritized compounds. The compound–target heatmap visualization of pXC_50_ activity data shown in Supplementary Material (Figure S6) highlighted four main active clusters including, cyclin-dependent kinase inhibitors (CDK), phosphatidylinositol-3-kinase (PI3K)/AKT/mammalian target of rapamycin (mTOR), epidermal growth factor receptor (EGFR) and serine/tyrosine checkpoint kinase inhibitors (CHEK). Activities are selective across the target panel as indicated by gray entries denoting potencies below the threshold.

**Figure 5:**
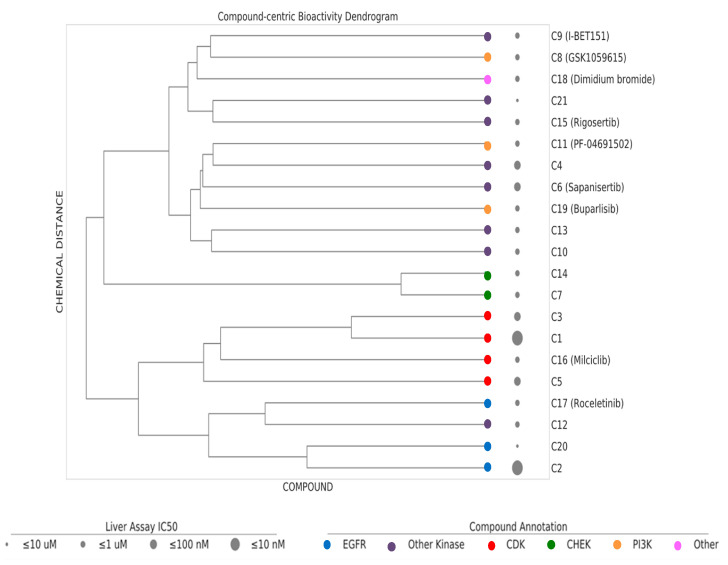
Dendrogram–hierarchical clustering depicts the hierarchical Ward’s clustering of 21 compounds using extended connectivity fingerprints with radius 4 (ECFP4). The compound nodes are colored based on their MoA (color coding). The drug panel of 21 compounds are shown as circles of increasing size proportional to the IC_50_ bioactivity in the hiPSC-Hep assay.

Based on dosage-dependent reduction in cellular lipid storage levels in the hiPSC-Hep, we performed dendrogram−hierarchical clustering to provide an unbiased view of 21 active compound hits from the annotated library screen. We selected compound-target links with bioactivity parameters, IC_50_ values, 10–1 µM, 1–0.1 µM, 0.1–0.01 µM and ≤ 0.01 µM. Compound molecular distances were clustered using the Ward’s method and the resulting structural similarity of the kinase inhibitors is visualized as dendrogram depicted in Figure 5. The clustering is consistent with mechanism of action (MOA) class annotations. One of the most active compounds was compound C1. Compound C16 and compound C3 were neighboring hits. These compounds were protective against the steatosis phenotype in hiPSC-Hep and cluster together in the dendrogram as belonging to the CDK family of kinases. We prioritized these three compounds for further analysis based on a combination of cutoff criteria including efficacy, concentration dependency, low cytotoxicity, and the presence of multiple structurally similar compounds for target validation.

**Figure 6:**
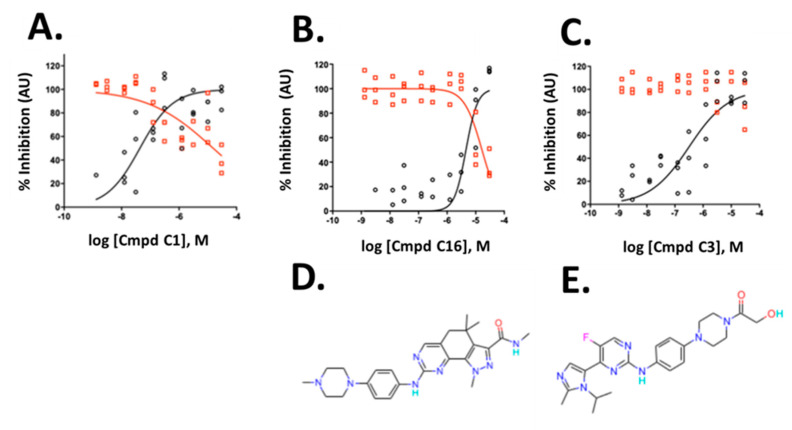
Validation of hits from the primary screen. (**A**–**C**) Dose-response data and non-linear curve fit of the data for integrated spot signal (black circles) and valid cell count (red circles) of three cyclin-dependent kinase inhibitors. (**D**,**E**) Compound structures of C16 and C3 are shown below the corresponding graphs**.** C1 structure is not shown**.** To allow verification of the data by independent means, AstraZeneca will make compound C1 available at no cost to an academic group or independent testing service for such validation testing. A small amount of compound will be provided, structure blind, under a material transfer agreement.

The three compounds exhibited IC_50_ values of 45 nM, 4.6 µM and 0.32 µM for compounds C1, C16 and C3, respectively, and affected viability only at high concentrations with IC_50_ values of 17.8 µM, 16.6 µM, and >30 µM for compound C1, C16 and C3, respectively (Figure 6A–C). The compounds belong to the CDK inhibitor family. Compound (C16) is Milciclib, a potent CDK2 inhibitor and compound C1 and C3 are likewise cyclin-dependent kinase inhibitors. Compound C3 is a reported anti-cancer compound with good oral exposure and has a similar piperazine scaffold to Milciclib (Figure 6D,E) [43]. Compound C1 is part of an active AstraZeneca program and its structure is not shown.

### 2.4. CDK Inhibitor Hits Modulate the C/EBP Target Gene Pathway

The CDK class of cyclin-dependent protein kinases has 13 members, CDK1–CDK13. The pXC50 values for C1, C16 and C3 against the CDK members are listed in Supplemental Table S1. Hit analysis from the screen specifically revealed our most potent hit, C1, to inhibit cyclin dependent kinases CDK5 >2 >1 >4 in order of potency. C3 exhibited potent inhibition against CDK 2 >1 >4 and C16 predominately targeted CDK 6 >2 >7 >4. Published studies indicate CDK2/4 activation is linked to development of NAFLD via regulation of the CCAAT/enhancer binding protein (C/EBP) α-histone acetyltransferase and recruitment of E1A binding protein P300 (EP300) [38,39]. We focused on the CDK pathway to confirm that the C/EBP-P300 signaling pathway was active in the hiPSC-Hep treated with TG-FA.

**Figure 7:**
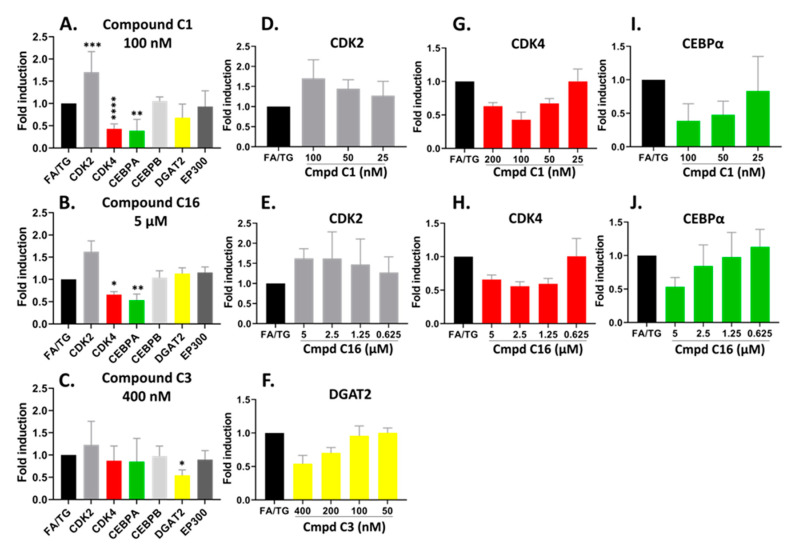
Confirmed hits modified the mRNA expression profile of CDK pathways by RT-qPCR analysis. hiPSC-Hep were treated for 18 h with TG/FA following pre-treatment in the presence or absence of C1, C16 and C3. The data are presented as an average fold-induction in the expression of each gene in the compound-treated cells relative to expression in the FA/TG treated cells, which was assigned the value 1 (black bar). The results showed downregulation of CDK4 (red bar) and CEBPA (green bar) gene expression and upregulation of CDK2 (gray bar) gene expression by compound C1 (**A,D,G,I**); downregulation of CDK4 (red bar) and CEBPA (green bar) gene expression by compound C16 (**B,E,H,J**) and downregulation of DGAT2 (yellow bar) gene expression by compound C3 (**C,F**). Significant difference from the TG/FA control group denoted by * *p* < 0.01, ** *p* < 0.005, *** *p* < 0.001, **** *p* < 0.0001 one-way analysis of variance (ANOVA) followed by post hoc comparisons of group means with the Tukey’s multiple comparison tests. Primers used are listed in Table 1.

To explore the signaling partners triggered or altered by the drugs, we performed gene expression RT-qPCR. The hiPSC-Hep were pre-treated in a 24-well plate with the three compounds shown in Figure 7 for 24 h followed by TG/FA challenge for an additional 18 h and RNA from each treatment well was isolated. The pre-treatment paradigm was chosen to stay consistent with how the original HTS was performed. We analyzed several target gene expression levels in the C/EBP-EP300 pathway including CDK2, CDK4, C/EBPα and C/EBPβ, EP300, and diacylglycerol acyltransferase 2 (DGAT2).

The compounds are potent CDK2 inhibitors (Table S1). The gene expression analysis of the CDK2 gene showed an overall increase in hiPSC-Hep treated with the compounds C1 and C16 at the 48 h treatment time (Figure 7A,B). There was a significant dose-response trend for C1, however, the data were not statistically significant for C16 (Figure 7D,E). On the other hand, the CDK4 gene was significantly downregulated by both compounds C1 and C16 at 100 nM and 5 µM, respectively (Figure 7A,B). These concentrations suppressed CDK4 gene expression to approximately 57% and 34% by compound C1 and C16, respectively. In addition, the inhibition of CDK4 showed dose-dependent trend with compound C1 from 25–100 nM (Figure 7G) and compound C16 (Figure 7H).

Next, to identify transcriptional changes in the pathways that arise from modulation of CDK2 and CDK4 gene expression, we sought to analyze gene expression of specific transcriptional regulators that could explain the observed phenotypic variation. Thus, we examined both C/EBPα and C/EBPβ gene expression, which has been implicated in the regulation of lipogenesis and activation of promoters of TAG-synthetic genes during development of hepatic steatosis [44,45,46]. The compounds did not show a significant effect on gene expression of C/EBPβ. On the other hand, we observed dose-dependent downregulation of the C/EBPα gene by compound C1 and C16 in parallel with downregulation of CDK4 (Figure 7I,J); C/EBPα mRNA expression was suppressed by 61% and 47% in the presence of 100 nM and 5 µM for compound C1 and C16, respectively. C/EBPα and C/EBPβ proteins regulate gene expression through the recruitment of EP300 to their promoters [47,48]. The EP300 gene expression was not significantly affected under our treatment conditions with compound C1 or C16.

As shown in Figure 7C, we observed the mRNA level of DGAT2, which encodes an enzyme that catalyzes the final step in TAG synthesis, was significantly decreased by compound C3 treatment in a dose-dependent manner by 46% under condition of hepatic steatosis (Figure 7C,F). In contrast to C1 and C16, compound C3 exhibited no effect on the mRNA levels of CDK2, CDK4 and EP300-C/EBPα/β pathway (Figure 7C).

## 3. Discussion

The discovery of novel therapeutic targets has been hindered by the lack of cell models that accurately reflect liver human disease pathology [19,49]. In regard to the NAFLD disease spectrum, in vitro models should recapitulate the transcriptional and metabolic dysregulations seen in fatty liver disease (NAFLD) and be amenable to HTS and mechanistic studies to develop new therapeutics [50]. Development of hiPSC-based models derived from iPSC cells from healthy individuals or from patients harboring single nucleotide polymorphisms (SNP) that contribute to a high risk for developing NAFLD/NASH, such as the I148M mutation in the patatin-like phospholipase domain containing 3 (PNPLA3) gene, offers the opportunity to study the cellular and molecular bases of NAFLD and to create robust cell platforms for drug discovery and translation to in vivo efficacy and toxicity [9].

There is currently a limited set of chemical compounds identified through high content assays in hiPSC-Hep [9,25,27,51] and none of these compounds affect steatosis in hiPSC-Hep models. Our high-throughput phenotypic assay platform that monitors quantitative accumulation of lipid droplets models ER stress-induced steatosis in hiPSC-Hep to recapitulate features of steatosis pathology and progression in NAFLD [15]. We showed that upon induction of steatosis, the expression of a panel of genes important for lipid metabolism is altered in the same manner as observed in liver biopsies from patients with high versus low levels of steatosis [34]. Treatment with the clinical candidate, obeticholic acid reversed the hepatic steatosis phenotype and transcriptomic analysis confirmed the selective activation of FXR target genes including upregulation of FGF19 and PLIN1 and downregulation of SLC51B and CYP7A1 genes by OCA. Additionally, our results obtained based on RNA-seq data were in good agreement with RT-qPCR data.

The design of our high content assay is suited for the screening of medium sized compound libraries. We collaborated with AstraZeneca Open Innovation Group and obtained a copy of their annotated library set of 13,000 bioactive compounds. The library was delivered with compounds dry spotted in 384-well screening plates requiring the need to pre-treat cells with drug. Our studies using OCA pre-treatment validated this approach for HTS. Here, we measured the number and intensity of “per cell” spots as an indicator of lipid droplet accumulation after treatment of test compound at 10 μM concentration to identify inhibitors of lipid accumulation. Cell viability parameters in the nuclear channel identify a cytotoxic compound. Our screen resulted in a 1.3% hit rate and 0.8% cytotoxic compounds. Dose-response studies revealed 21 compounds that inhibited ER-stress induced lipid droplet accumulation with EC_50_ ≤ 10 μM and IC_50_ for cell viability > five-fold.

Clustering analyses of the screening hits resulted in identification of CDK2/4 and DGAT2 inhibitors as the predominate modulators of the hiPSC-Hep ER-stress induced lipid accumulation phenotype. Interestingly, these targets were previously identified as priority drug targets for prevention and treatment of hepatic steatosis [37,38,39,52]. CDK4 is a critical regulatory enzyme and patients with steatosis and NASH have increased levels of CDK4 [38,39]. Targeting CDK4 in aged mice by a specific CDK4 inhibitor eliminates hepatic steatosis. C/EBPα belongs to a transcription factor family of six members which are involved in a variety of cellular responses and regulates hepatic lipid metabolism by promoting the expression of lipogenic genes by inducing the expression of PPARγ [45]. C/EBPα is the main target of CDK4 [53,54] which mediates phosphorylation of C/EBPα at Ser193 and stimulates C/EBPα/histone acetyltransferase p300 (p300) complex formation and increases steatosis in mice [37,38]. Thus, targeting CDK4 and C/EBPα could be an ideal drug development strategy.

Compound C3 was the only compound of the three to significantly inhibit expression of DGAT2. We chose to test gene expression of DGAT2 because compound inhibition of cdk4 activity decreased expression of predominately DGAT2 proteins in mouse livers under high fat diet conditions [38]. We know the pathway to TAG synthesis via DGAT2 is active in hiPSC-Hep, since triacsin C completely inhibits lipid accumulation induced by exogenous oleic acid. Moreover, we showed OCA significantly inhibited DGAT2 expression after 4 and 18 h treatment with thapsigargin and free-fatty acids [34]. Thus, pharmacological inhibition of DGAT proteins, which play a critical role in triglycerides production as well as lipolysis and lipotoxic stress [55], may be a starting point to develop a novel class of DGAT2 inhibitors.

Our screen successfully identified drug targets whose mechanism of action potentially contributes to the underlying fatty liver state in hiPSC-Hep. The identification of these drugs with known mechanisms of action that act as modulators of ER-stress induced lipid accumulation in hiPSC-Hep reveals novel biology that could provide new opportunities in facilitating identification of novel drug targets for the prevention and treatment of NAFLD. Further studies will include characterization of these drug targets on hiPSC-Hep derived from patients with the I148M mutation in the patatin-like phospholipase domain-containing protein 3 (PNPLA3) gene, a major genetic risk factor for NAFLD [56,57,58,59]. We have obtained these cells as a collaboration with Fuji Film Cellular Dynamics. These patient specific hepatocytes have shown increased lipid accumulation in the absence of exogenous fatty acid and we will evaluate whether the drug targets identified from our HTS campaign reverse the phenotype in PNPLA3 hiPSC-Hep.

## 4. Materials and Methods

### 4.1. Cell Culture and Differentiation of hiPSC to Hepatocytes

Cryopreserved hiPSC-derived hepatocytes (iCell Hepatocytes 2.0; Cellular Dynamics International Madison, WI) plated at a density of 36 K/well in 384-well on collagen I coated Corning Biocoat 384-well plates (BD Biosciences, 101306), were cultured to promote hepatic differentiation and maturation into hepatic-like cells as previously described [34]. Hepatocytes were plated according to the manufacturer’s protocol in a volume of 50 µL/well using BioTek^TM^ Microflo^TM^ select dispenser (#7170011, BioTek Instruments) for high-throughput and high-content analysis assay (HT/HCA) drug discovery. After plating cells were cultured according to vendor specifications in a cell culture incubator at 37 °C, 5% CO_2_ for 3–4 h. Then, 40 µL spent medium was aspirated using multi-channel automated pipette handling system (Integra ViaFlo 96/384) and replaced with 40 µL (80% exchange) of fresh plating medium. Approximately 24 h post-plating 80% of medium was replaced every day thereafter. At day 5 of differentiation, 40 µL/well spent medium was replaced with maintenance medium (MM) consisting of William’s E medium (Thermo Fisher Scientific) supplemented with 0.1 µM dexamethasone and hepatocyte maintenance supplement pack (Thermo Fisher Scientific) for an additional 2–3 days before HCA. The overview of automated HT system for hiPSC-derived hepatocytes differentiation, staining and image analysis for phenotypic screening is depicted in Figure S2. Primary screening hit confirmation and dose response studies were performed on different batches of hiPSC-derived hepatocytes.

### 4.2. TAG Accumulation, Staining and High-Content Imaging

To induce hepatic triglycerides (TAG) accumulation, before staining, cells were treated with 20 μL of 2× FA mixture, consisting of different proportions of oleic acid (OA-BSA, 25 µM, Sigma, #O3008), palmitic acid (PA-BSA, 200 µM, Agilent Technologies, #102720100) and thapsigargin (TG, 1 µM, Abcam, #120286) with a total volume of 40 μL/well. The TG/FA mixture was dispensed with BioTek^TM^ Microflo^TM^ select dispenser (#7170011, BioTek Instruments) and incubated for 18 h. Next, to label TAG accumulation stored into cytoplasmic lipid droplets, upon treatment, living cells were washed two times with warm Dulbecco’s Phosphate-Buffered Saline 1X (PBS, Corning, #21-031CV) with a handheld 384-channel electronic pipette device and then incubated for 15 min with a lipophilic dye, BODIPY 493/503 (8 ug/mL Life technologies, #D3922-green), in PBS at 37 °C. BODIPY (493/503) has become the standard dye utilized by scientists to study lipid droplets in live cells [60]. After three washes with PBS cells were fixed with 4% paraformaldehyde methanol-free (PFA, Electron Microscopy Science, #15710) in PBS for 15 min at room temperature (RT). Cell health was monitored by Hoechst 33342 (10 ug/mL, Molecular Probe, #H21492) staining in PBS for nuclear count. After TAG staining the 384-assay plates were loaded in an automated confocal microscope with Opera High Content Imaging System (PerkinElmer, Waltham, MA, USA). Dyes were excited and images were captured on the Hoechst 3342 and Alexa Fluor 488 channels and signals were collected with a long pass filter 410 and 505 nm, for nuclei and TAG, respectively, at objective 20× air objective for HCA. To capture enough cells (>600) for the analysis, eight fields per well were imaged. Hoechst 33342 fluorescence was used to identify the nuclear region and cell counts. To assess intracellular fat deposits, BODIPY 493/503 fluorescence intensity was measured inside the cytoplasm region. The experimental conditions were determined in advance by performing dose curves and by assessing cell viability which was always comparable to control cells (100% viability) treated with the equivalent concentrations of FA-free bovine serum albumin (BSA, Sigma, #A6003) and the vehicle (0.1% DMSO), as previously described [34].

### 4.3. Compound Library

The compound library, consisting of 13,000 compounds arrayed in 384-well plates as single compounds at 10 mM in DMSO, used in the screen was the annotated collection set (AstraZeneca Open Innovation, https://openinnovation.astrazeneca.com/target-innovation.html). The agreement between Sanford Burnham Prebys (SBP) Medical Discovery Institute and AstraZeneca gave us access to annotated molecules suitable for phenotypic assay target screening and deconvolution, including compounds with an affinity of less than 100 nM on 1763 human targets. The quality of all compounds was assured by AstraZeneca as greater than 90% pure, with provided quality control data. The library in the primary screen was screened at a constant 1:1000 dilution, with a 10 µM final concentration of compound in each well (0.1% DMSO) in the primary screening.

### 4.4. 13000-Compound Library High-Content Screening

A screen was performed using a 384-well hiPSC-hep platform optimized for phenotypic screening on a highly annotated phenotypic library collection of 13,000 molecule compounds arrayed individually in 36 384-well plates, provided by the AstraZeneca Open Innovation to identify inhibitors of fatty acid and endoplasmic reticulum (ER) stress-induced lipid droplet accumulation. All AstraZeneca pre-spotted 384-well source plate compounds were dissolved in 100% DMSO as 10 mM stock solutions and stored at −80 °C before use in all subsequent experiments. Compound library was selected based on known compound–target interactions extracted from bioactivity databases. The library for the phenotypic screen can be explained by the hypothesis that compounds that were previously found to be active are more “hit-like” than other molecules and are therefore more likely to show bioactivity in the phenotypic screen. The 36 384-well plates representing the library were screened over a 2-month period (Figure S3).

For the HCA screen, compounds tested for their inhibitory effects, were used as single dose at a 10 μM final concentration of compound in DMSO in each well (0.1% DMSO) in 20 μL of maintenance medium. Prior to compound addition, 30 µL/well of medium was removed from hiPSC-Hep cells screening plates with ViaFlo (10 µL/well remaining). Pre-spotted 80 nL of 10 mM compound were diluted to 20 μM (0.2% DMSO) in 40 μL of maintenance medium in the source 384-well plate. Next, 10 μL were added directly with ViaFlo to the destination plate containing hiPSC-Hep cells in 10 μL of maintenance medium and pre-incubated for 18 h at 37 °C, 5% CO_2_. Then, 20 μL of a mixture of FA and TG was added with BioTek for an additional 18 h to the cells pre-treated with compounds to a final assay volume of 40 µL/well. Sterile metal lids were used to shield the assay wells from the edge effects of evaporation or water uptake. In total, assay plates were incubated for 36 h prior to fixation and BODIPY staining. Positive and negative controls for the screen included BSA containing only 0.1% DMSO (positive control for inhibition) and a mixture of FA and TG (negative control for inhibition), to induce the steatotic phenotype, which were located in columns 11 and 12 of the plate, respectively. The rest of each plate (row A to P, columns 1 to 24) was used for compound screening. The plate layout and library overview are depicted in Figure S3. Our screening was performed in three batches and to avoid normalization issues, we included controls in each plate to monitor variation in background so that hits could be computed and identified independently. The solutions were dispensed using automated liquid handling devices as depicted in Figure S2.

### 4.5. Secondary Hit Confirmation and Target Validation

To confirm hits obtained from the primary screen and validate their inhibitory effects on lipid droplet accumulation, compounds were re-tested in the secondary screen at two different concentrations (1 and 10 μM). The compound effects were derived from three independent experiments, three separate plates, and Z’ factor were determined. Follow-up assays were carried out to confirm the activity of compounds that scored as “positives” in the secondary screen. Those giving a reproducible activity were used for compound validation tested in the confirmation screen to produce 6-point dose–response curves ranging from 10 µM to 0.625 µM, with triplicate sampling. Active compounds of interest for further study were defined as those with a reproducible IC_50_ of less than 5 µM. These compounds were subjected to further analysis including cytotoxicity measurements and molecular target identification. In addition, AstraZeneca Open Innovation provided liver assay IC_50_ and pXC50 activity data of hits against various targets.

### 4.6. HTS Data-Processing and Hit Identification Methods

By analyzing Opera confocal microplate imaging system (PerkinElmer, Inc., Waltham, MA) acquired images with a proprietary algorithm developed in the Acapella^®^ 2.6/2.7 High Content Image Analysis software package (PerkinElmer) and executed on Columbus High Content image data management system (PerkinElmer), we were able to find nuclei and to track and quantify individual lipid droplet spots. All images were acquired with a 20x air objective. Six fields were acquired per well. For quantitative analysis individual hiPSC-Hep were segmented based on the Hoechst 33342 nuclear stain using the “Find Nuclei” building block and using the “Select Cell Region” building block, a ring region with a width of 15 pixels was then created around the individual nuclei that was further masked to classify them as cells. Individual BODIPY-stained lipid droplets dispersed as spots throughout the cytoplasm inside cell region were analyzed using “Select Cell Region” and ”Find Spots” building blocks (Figure S2). Then the properties of spots spaced less than 2 pixels were assessed in the cell population for a variety of parameters (e.g., intensity, number, area of spots, etc.) and analyzed. The images were further subjected to HCA to quantify the changes in the steatotic phenotype. The calculated parameters of the assay included cell count per well and well averages of “per-cell” values of nucleus area, roundness, and average and integrated Hoechst 33342 intensities, as well as integrated lipid droplet area and average and integrated BODIPY-green fluorescence intensities of the lipid droplet regions in the cytoplasm/cell. The well average of the integrated BODIPY-green fluorescence intensity of the lipid droplet region in the cytoplasm was selected as the primary assay readout since it correlated with the overall uptake of TAG per cell.

Data generated in Columbus were exported to CBIS software (ChemInnovation Software, Inc., San Diego, CA, USA) for normalization and hit identification. Edge effects and systematic dispensing patterns across the plates in the pilot screen were corrected with Genedata Screener^®^ Assay Analyzer 10.0.1 (Genedata, Basel, Switzerland) to prioritize compound sets for follow-up work. The robustness and reproducibility of the assay signal window are the most critical features of a HTS assay. We used a procedure to measure and evaluate the signal windows of our BODIPY staining HC assay by capturing data from Max and Min controls tested in every plate in each batch conducted on separate days. The assay performance was evaluated in terms of signal-to-background (S/B), statistical robustness was assessed by Z-factor and percentage covariance (%CV) of Max and Min signals. Z-factors between 0.5 and 1 predicted good performance in HCA. The assay used in the present study was developed and implemented in a 384-well plate format with the same layout for plate controls and compounds.

### 4.7. Quantitative Real-Time PCR

RNA was isolated from hiPSC-derived hepatocytes using the RNeasy kit (Qiagen, Frederick) and subsequently subjected to DNase digestion (Qiagen). Total RNA was quantified using NanoDrop 8000 (Thermo Scientific). The quality of total RNA was assessed by the Agilent Bioanalyzer Nano chip (Agilent Technologies) and RNA integrity number (RIN) ranged from 9.7 to 10. Reverse transcription was performed using the high capacity cDNA reverse transcription kit (Applied Biosystems, Foster City, CA, USA) according to the manufacturer’s recommendation and the complementary DNA (cDNA) obtained used for real-time quantitative PCR. The reaction mixture (10 μL), containing 1 μL of cDNA template, 0.5 μL each of primer and probe (20×, Thermo Fisher Scientific) mix for the gene of interest plus the reference gene and TaqMan Universal PCR master mix (Applied Biosystems) was added to the opaque white 384-well plates and amplified as follows: after 2 min of initial incubation at 50 °C and 10 min of initial denaturation at 95 °C, 45 cycles at 95 °C for 15 s, 60 °C for 1 min for annealing and 1 sec at 72 °C for final extension were performed. Assays were designed to have primers/probes to span exon–exon junctions (Table S1). Direct detection of PCR products was monitored by measuring the fluorescence produced by the result of TaqMan probe hydrolysis after every cycle. All experiments were carried out in triplicate in a LightCycler 480 thermocycler (Roche Diagnostic). Relative gene expression from qPCR data between groups of samples were assessed using a ∆∆Cq method [61]. To adjust for variations in the cDNA synthesis each gene was normalized to that of hypoxanthine phosphoribosyl transferase 1 (HPRT1] or GAPDH mRNA.

### 4.8. Dendrogram–Hierarchical Clustering Using Ward’s Method

Chemical similarity assessment was performed for the set of 21 compounds from the AstraZeneca library that were prioritized after testing in the high-content screening assay. Extended connectivity fingerprints with a depth of 4 bonds (ECFP4] were used to represent the compound chemical structures [61] and pairwise distances were computed. Ward’s hierarchical clustering was performed with the generated distance matrix as the input [62]. Ward’s minimum variance method is an agglomerative clustering algorithm where the error sum of squares objective function is applied recursively to merge compound clusters. This method groups the compounds using a “score” obtained by measuring the average of the chemical distance between them. The score reflects the similarity/non-similarity between compounds and helps to identify active compounds. The output was graphically represented in a dendrogram tree representation that allows depiction of the merge order and distances during hierarchical clustering. A cluster is defined as two of more leaves that are joined by a common node and horizontal lines (branches) correspond to cluster merges. The vertical lines show the clusters/leaves that were merged to form a new cluster, the height of the branches depict the cluster distances and leaves represent the compounds. The clustering and the dendrogram were generated with the Python (3.6) SciPy (https://www.scipy.org/) and Matplotlib libraries (https://matplotlib.org/).

### 4.9. pXC50 Activity Annotations for Target Profiling

Activity annotations for the 21 prioritized compounds were collected from AstraZeneca data repository that stores both internal assay data, as well as those reported in external vendor and public databases (ChEMBL or PubChem). Precomputed log activities were used for activity data obtained from external sources. For consistency, internal biochemical assay endpoints viz. IC_50_, EC_50_, Ki, Kd and other measurements were also converted into a median log value for each compound target pair. The highest activity value was reported for a compound with multiple measured activity for a given target. Since, the log activity data consisted of different assay endpoint types, the activity annotations have been referred to as “pXC50”.

### 4.10. Statistical Analyses and Target Selection

All data were analyzed using GraphPad Prism 5.01 software (La Jolla, CA, USA). Non-linear regression analysis and curve fitting parameter were performed to calculate EC50s (GraphPad Software, La Jolla CA, USA) using dose–response curves for the compounds. Error bars of dose–response curves represent the standard deviation of three replicates. Z’ factor and EC50s were used as parameters for assay validation. Assay performance was routinely addressed in terms of signal-to-background (S/B) and Z′ factor (Z′). The Z’-factor, used to quantify the suitability of the lipid droplet assay for the high-throughput screen (HTS), was calculated based on the following equation:

Z’ = 1 − (3 SD of sample group + 3 SD of negative control)/(mean of sample group−mean of negative control). FA/TG-treated wells were defined as general sample group and BSA-treated wells were defined as negative controls for calculating Z’-factor. In our assay, the Z’ criteria were as follows: Z’ = 0.5–1.0 corresponds to an excellent assay; Z’ = 0–0.5 corresponds to a suboptimal assay; Z’ < 0 corresponds to an unsuccessful assay. Percent inhibition of lipid accumulation was calculated based on the positive (BSA-treated) and negative (FA/TG-treated) controls on each plate: % inhibition = 100 * [(compound value–AVEneg)/(AVEpos–AVEneg)]. RNA-seq data were analyzed by iPathwayGuide (Advaita Bioinformatics: http://www.advaitabio.com/ipathwayguide.html) as described in [35]. Differences between two groups were analyzed using Student’s *t*-tests. Fold-change expression values obtained from RT-qPCR were analyzed by one-way analysis of variance (ANOVA) followed by post hoc comparisons of group means with the Tukey’s multiple comparison tests at an overall confidence level of 95% using Prism software (GraphPad).

## Figures and Tables

**Table 1:** Genes used in the RT-qPCR analysis and their corresponding TaqMan gene expression assay IDs.

Gene	Assay ID	Exon Boundary Range	Amplicon Length
CDK2	Hs01548894_m1	2–3	58
CDK4	Hs00262861_m1	7–8	75
CEBPA	Hs00269972_s1	1–1	77
CEBPB	Hs00942496_s1	1–1	140
DGAT2	Hs01045913_m1	6–7	69
EP300	Hs00914223_m1	29–30	85

## Data Availability

Transcriptome data from this study are available at GEO under accession number GSE116185.

## Supplementary Materials

Supplementary materials can be found at https://www.mdpi.com/1422-0067/21/24/9557/s1.

## Abbreviations

NAFLD	Non-alcoholic fatty liver disease
NASH	Non-alcoholic steatohepatitis
TAG	triglycerides
HTS	high-throughput screening
HCS	high-content screening
HCA	high-content analysis
BSA	bovine serum albumin
OA	oleic acid
PA	palmitic acid
TG	thapsigargin
FA	free fatty acid
S/B	signal-to-background ratio
Z’	Z’-factor coefficient
CDK2	cyclin D3-cyclin-dependent kinase 2
CDK4	cyclin D3-cyclin-dependent kinase 4
CEBPA	CCAAT enhancer binding protein alpha
CEBPB	CCAAT enhancer binding protein beta
DGAT2	diacylglycerol O-acyltransferase 2
EP300	α-histone acetyltransferase E1A Binding Protein P300
