# Supplementary material for: Pluripotent Stem Cell-Derived Hepatocytes Phenotypic Screening Reveals Small Molecules Targeting the CDK2/4-C/EBPα/DGAT2 Pathway Preventing ER-Stress Induced Lipid Accumulation
Source: Int J Mol Sci. 2020 Dec 15;21(24):9557. doi: 10.3390/ijms21249557 (PMC7765409; doi:10.3390/ijms21249557)
Supplement: Supplementary file 1 [file ijms-21-09557-s001.pdf]

International Journal of *Molecular Sciences*

MDPI

Article

Title

Pluripotent Stem Cell-derived Hepatocytes Phenotypic Screening Reveals Small Molecules Targeting CDK2/4-C/EBPα/DGAT2 Pathway Preventing ER-Stress Induced Lipid Accumulation.

Maddalena Parafati [1, 2], Sang Hyo Bae [1], R. Jason Kirby [2], Martina Fitzek [4], Preeti Iyer [5], Ola Engkvist[5], David M. Smith [6] and Siobhan Malany [1, 2, *]

Supplementary Materials:

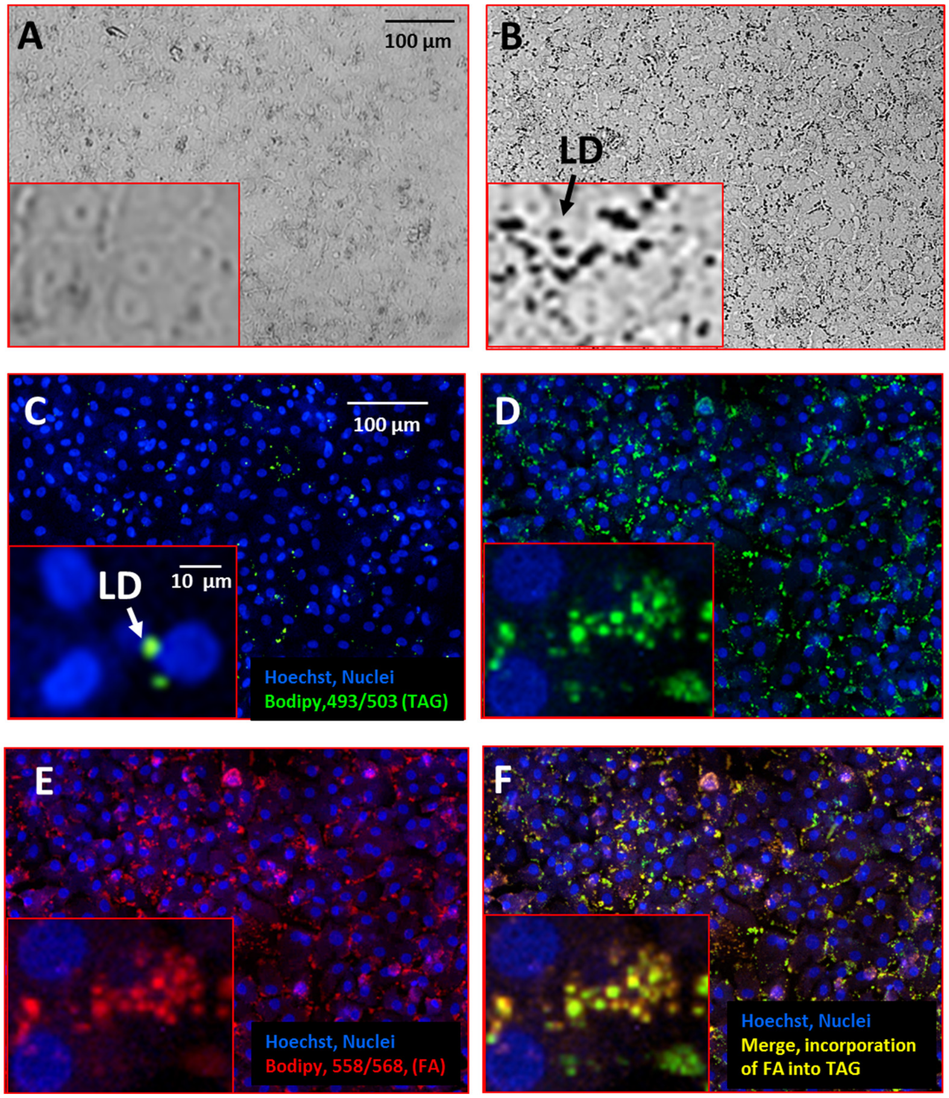

Figure S1. Accumulation of lipid droplet in hiPSC-Hep visualized by conventional light and fluorescent microscopy. The high refractive index difference of neutral lipids compared to cytoplasm can be used to visualize lipid droplets in control (**A**) and cells exposed to fatty acid cocktail (**B**) by conventional light microscopy. Detection of lipid droplet stained with BODIPY 493/503 dye shows TAG accumulation (green) in control cells (**C**) and in cells treated with thapsigargin and fatty acid mixture (**D**)**.** For fatty acid uptake and trafficking, hiPSC-Hep were labeled for 18 h with 2 µM BODIPY 558/568 C_{12}, a saturated fluorescent fatty acid analog (red) (**E**). Co-localization of both BODIPY 493/503 and BODIPY 558/568 indicates incorporation of fatty acid analog into lipid droplet-specific neutral lipids (yellow) (**F**). Fixed cells were stained with Hoechst dye (blue) for nuclei and imaged with a 20x objective. An enlarged region of interest is shown at the bottom left corner in each figure.

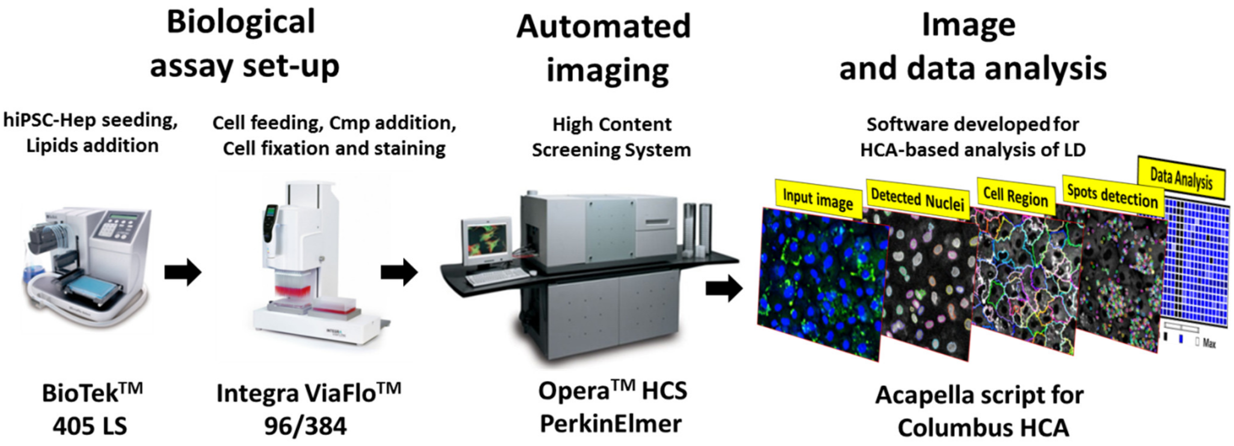

Figure S2. Overview of automated high-throughput system for phenotypic screening in hiPSC-Hep. The assay consists of five stages including hiPSC-Hep seeding and differentiation, compound and lipid addition, staining and image acquisition and analysis. Cellular responses are extracted from images and used to construct a phenotypic profile (right; cartooned in two dimensions). Four main steps of image analysis storage, segmentation of objects (nuclei, whole cell and lipid droplet).

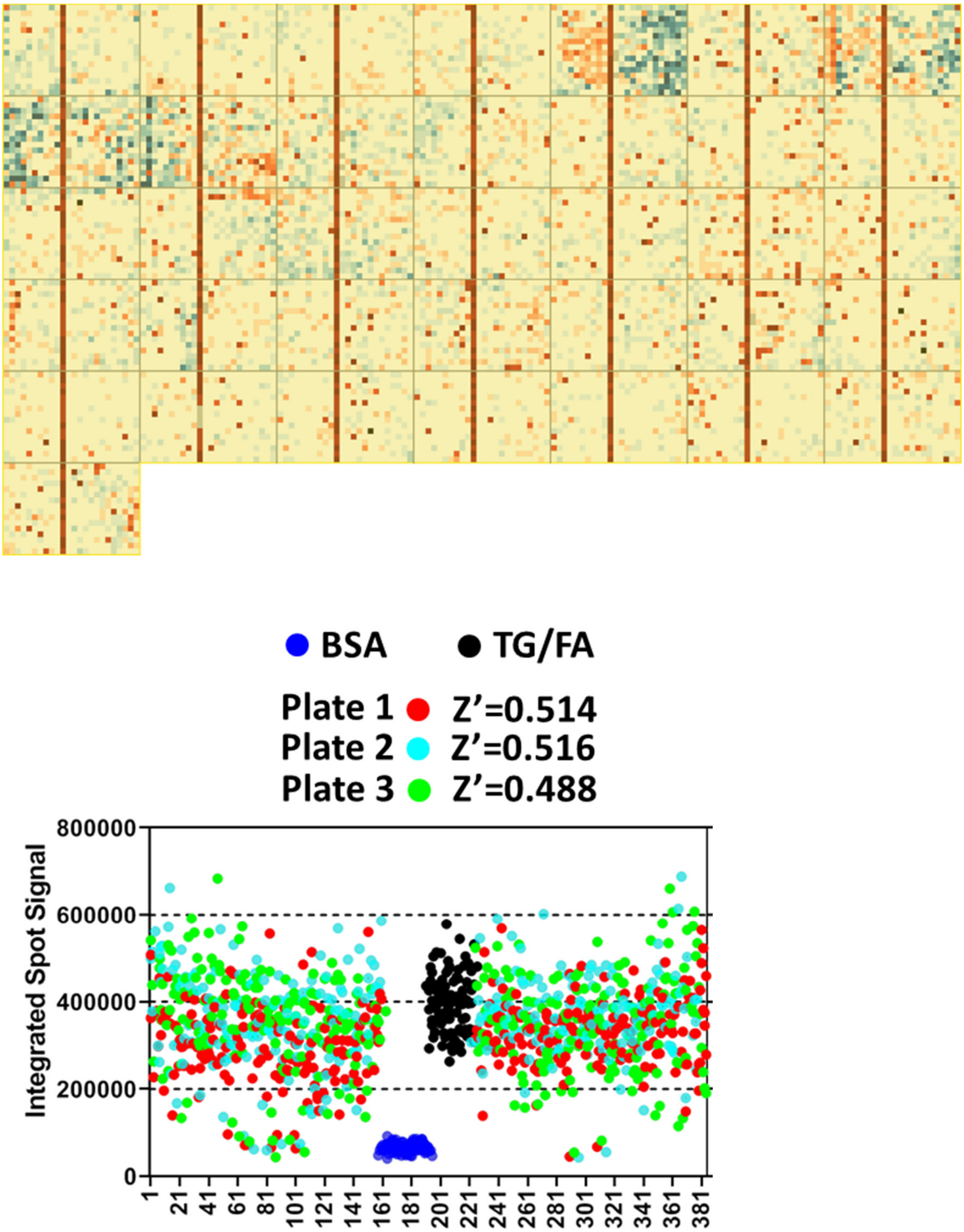

Figure S3. AstraZeneca Annotated Library Screen overview. Assay overview of 36 plates during the AstraZeneca screening in hiPSC-Hep (upper figure). The heatmaps show Astra Zeneca library compound dispensing layout in a 384-well assay plate. Red indicates 100% percent inhibitory activity based on average of control wells response, Blue indicates -100% response and yellow indicates 0% response. In each plate controls are located in the center with sixteen positive controls (BSA, column 11) and sixteen negative controls (TG/FA, column 12). The BSA only column 11 is shown by the solid red stripe. Confirmation of screening hits performed in triplicate across three plates is shown in the lower figure. Blue dots are BSA treated wells and black dots are TG/FA treated wells.

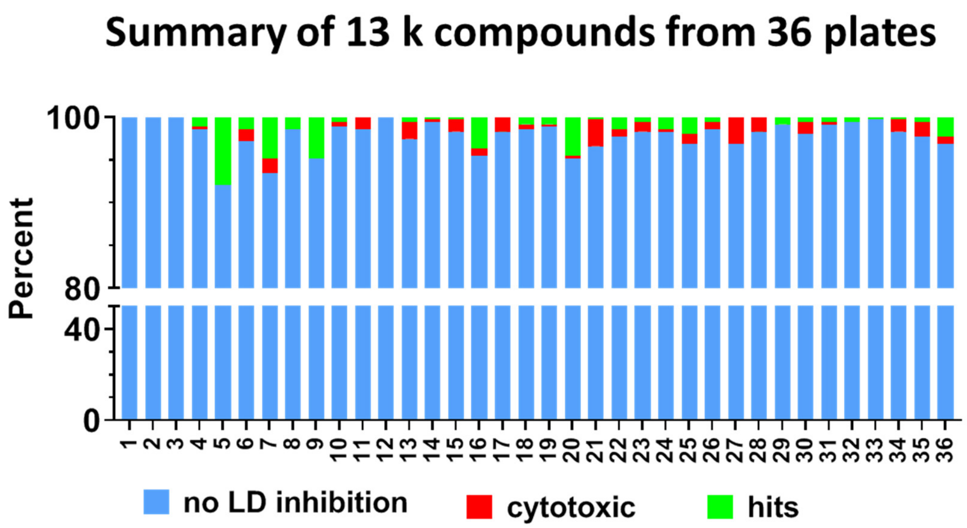

Figure S4. Summary of library screen and hit confirmation. Relative number of wells showing no effect (blue), ≥ 50% reduction in ISS (green) and < 60% valid cell count or cytotoxic (red) per screening plate.

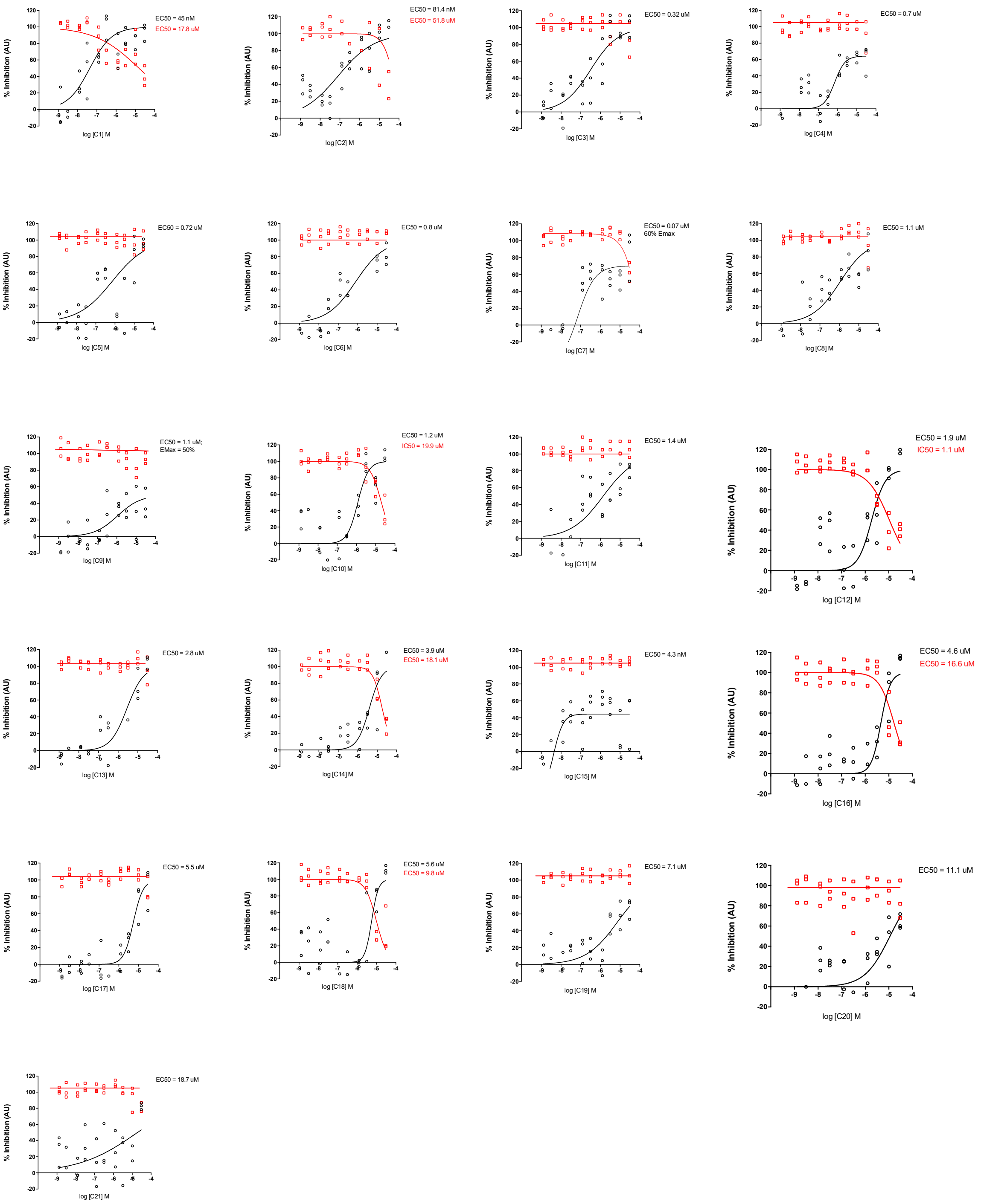

Figure S5. Dose-response activity for 21 hits (C-C21) in the hiPSC-Hep on inhibition of lipid droplet accumulation (black line: ISS) and on viable cell count (red line). EC_{50} values are noted in graphs.

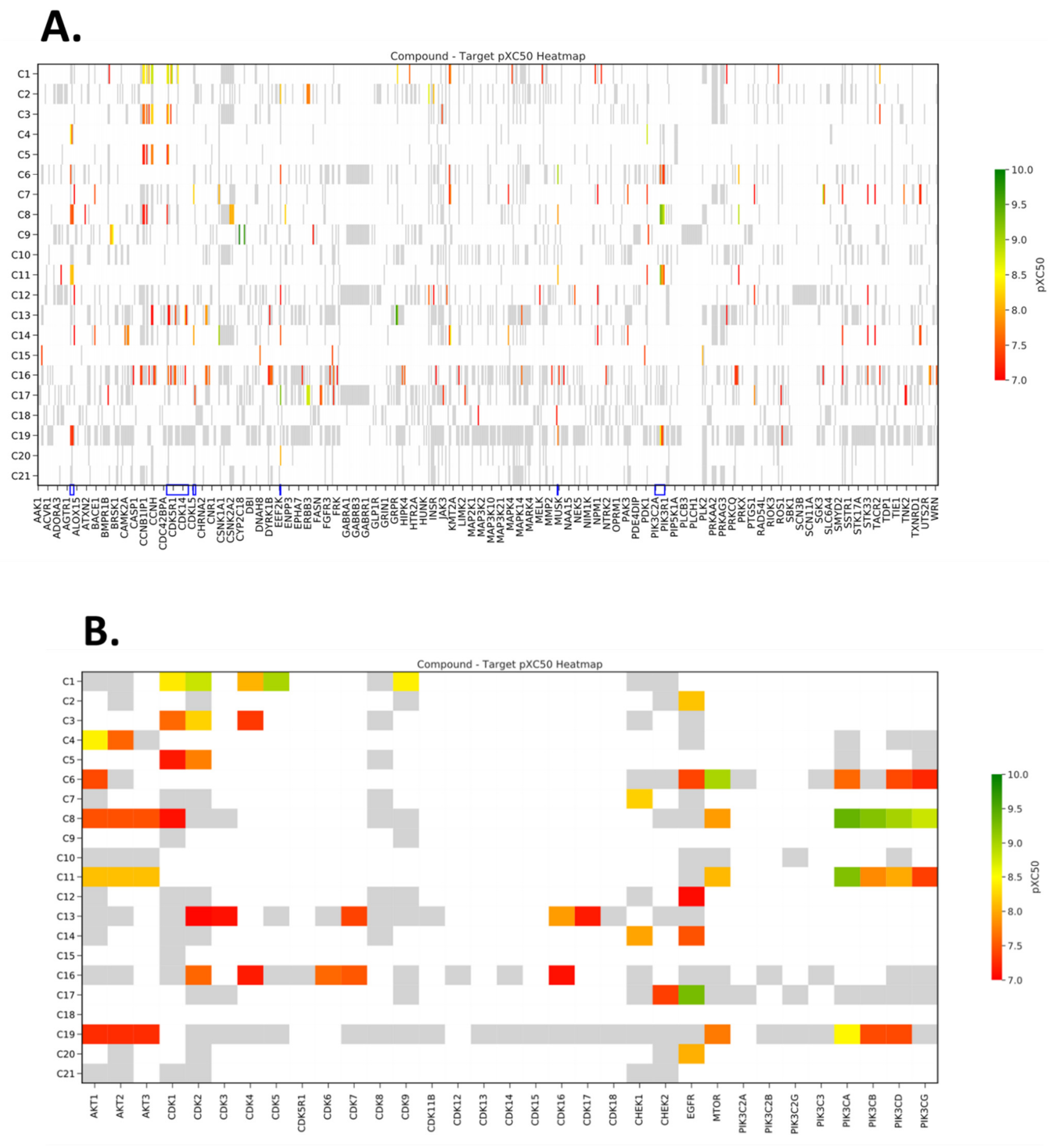

Figure S6. (**A**) A compound-target heatmap for visualization of pXC50 activity data of hits against various targets. pXC50 values were obtained as outlined in Material and Methods Section 4.9. Targets and compounds are plotted along the x- and y-axis respectively. An activity threshold of pXC50 = 7.0 has been applied and entries are depicted according to the color bar. Gray cells indicate target activity values below the threshold and white cells denote missing data. Compounds on the y-axis are sorted by decreasing potency in the hiPSC-Hep assay. Blue rectangles/lines along the x-axis highlight the four gene clusters. The heatmap was generated using Python Seaborn library (https://seaborn.pydata.org/). (**B**) A subplot of the heatmap showing the pXC50 activity data of hits against the 4 target clusters highlighted in (a).

Table S1. In-Vitro Binding data for hits and their corresponding ID

Sample ID	EGID	Target	Gene Name	pXC50
C3	1017	CDK2	cyclin dependent kinase 2	8.24
C3	983	CDK1	cyclin dependent kinase 1	7.61
C3	1019	CDK4	cyclin dependent kinase 4	7.33
C3	1024	CDK8	cyclin dependent kinase 8	5.60
C1	1020	CDK5	cyclin dependent kinase 5	9.00
C1	1017	CDK2	cyclin dependent kinase 2	8.82
C1	983	CDK1	cyclin dependent kinase 1	8.38
C1	1019	CDK4	cyclin dependent kinase 4	8.06
C1	1025	CDK9	cyclin dependent kinase 9	7.25
C1	1024	CDK8	cyclin dependent kinase 8	5.60
C16	1021	CDK6	cyclin dependent kinase 6	7.61
C16	1017	CDK2	cyclin dependent kinase 2	7.60
C16	1022	CDK7	cyclin dependent kinase 7	7.51
C16	1019	CDK4	cyclin dependent kinase 4	7.16
C16	5127	CDK16	cyclin dependent kinase 16	7.10
C16	983	CDK1	cyclin dependent kinase 1	6.84
C16	1020	CDK5	cyclin dependent kinase 5	6.58
C16	8851	CDK5R1	cyclin dependent kinase 5 regulatory subunit 1	6.58
C16	5218	CDK14	cyclin dependent kinase 14	6.13
C16	1025	CDK9	cyclin dependent kinase 9	5.60
C16	51755	CDK12	cyclin dependent kinase 12	5.34

© 2020 by the author. Licensee MDPI, Basel, Switzerland. This article is an open access article distributed under the terms and conditions of the Creative Commons Attribution (CC BY) license (http://creativecommons.org/licenses/by/4.0/).
